# Nlrp6 promotes recovery after peripheral nerve injury independently of inflammasomes

**DOI:** 10.1186/s12974-015-0367-8

**Published:** 2015-08-08

**Authors:** Elke Ydens, Dieter Demon, Guillaume Lornet, Vicky De Winter, Vincent Timmerman, Mohamed Lamkanfi, Sophie Janssens

**Affiliations:** Peripheral Neuropathy Group, Department of Molecular Genetics, VIB and University of Antwerp, Universiteitsplein 1, B-2610 Wilrijk, Antwerpen Belgium; Neurogenetics Laboratory, Institute Born-Bunge and University of Antwerp, Universiteitsplein 1, B-2610 Antwerpen, Belgium; Department of Medical Protein Research, VIB, Gent, Belgium; Department of Biochemistry, Ghent University, Gent, Belgium; Unit Immunoregulation and Mucosal Immunology, GROUP-ID Consortium, VIB Inflammation Research Centre, Technologiepark 927, B-9052 Gent, Belgium; Department of Internal Medicine, Ghent University, Gent, Belgium

## Abstract

**Background:**

NOD-like receptors (Nlrs) are key regulators of immune responses during infection and autoimmunity. A subset of Nlrs assembles inflammasomes, molecular platforms that are activated in response to endogenous danger and microbial ligands and that control release of interleukin (IL)-1β and IL-18. However, their role in response to injury in the nervous system is less understood.

**Methods:**

In this study, we investigated the expression profile of major inflammasome components in the peripheral nervous system (PNS) and explored the physiological role of different Nlrs upon acute nerve injury in mice.

**Results:**

While in basal conditions, predominantly members of NOD-like receptor B (Nlrb) subfamily (NLR family, apoptosis inhibitory proteins (NAIPs)) and Nlrc subfamily (ICE-protease activating factor (IPAF)/NOD) are detected in the sciatic nerve, injury causes a shift towards expression of the Nlrp family. Sterile nerve injury also leads to an increase in expression of the Nlrb subfamily, while bacteria trigger expression of the Nlrc subfamily. Interestingly, loss of Nlrp6 led to strongly impaired nerve function upon nerve crush. Loss of the inflammasome adaptor apoptosis-associated speck-like protein containing a CARD (ASC) and effector caspase-1 and caspase-11 did not affect sciatic nerve function, suggesting that Nlrp6 contributed to recovery after peripheral nerve injury independently of inflammasomes. In line with this, we did not detect release of mature IL-1β upon acute nerve injury despite potent induction of pro-IL-1β and inflammasome components Nlrp3 and Nlrp1. However, Nlrp6 deficiency was associated with increased pro-inflammatory extracellular regulated MAP kinase (ERK) signaling, suggesting that hyperinflammation in the absence of Nlrp6 exacerbated peripheral nerve injury.

**Conclusions:**

Together, our observations suggest that Nlrp6 contributes to recovery from peripheral nerve injury by dampening inflammatory responses independently of IL-1β and inflammasomes.

## Introduction

Interleukin-1 (IL-1) was the first cytokine described to act on the brain. It exerts multiple actions on the nervous system including induction of fever, suppression of appetite, and modulation of sleep, as well as alterations in immune or neuroendocrine functions [1]. While IL-1 is not neurotoxic to neurons in culture or upon injection in a normal healthy brain [2], it exacerbates neuronal death and damage caused by other insults such as ischemia, trauma, or excitotoxic injury [2, 3]. IL-1 expression levels are strongly elevated in several conditions of acute injury to the central nervous system (CNS), just like in human neurodegenerative patients [4]. Inhibition of IL-1 in vivo by using blocking antibodies, the IL-1 receptor antagonist IL-1ra, or genetic mouse models for caspase-1 deficiency leads to a dramatic reduction in neuronal loss upon stroke or brain trauma [5, 6]. As such, IL-1 has been postulated as a major culprit in acute neurodegeneration [1, 5].

The term interleukin-1 refers to two closely related family members, IL-1α and IL-1β, that are both synthesized as precursor proteins [7]. Unlike pro-IL-1α, pro-IL-1β is strictly dependent on processing by caspase-1 to gain biological activity [7]. Processing occurs in a molecular platform, called the inflammasome [8–10]. Inflammasomes are assembled around a pattern recognition receptor (PRR) molecule that belongs to the NOD-like receptor (Nlr) or HIN-200 protein family [11]. Upon ligand binding, they recruit an adaptor protein apoptosis-associated speck-like protein containing a CARD (ASC) that serves as a scaffold for recruitment, oligomerization, and autoprocessing of caspase-1 [10]. Several inflammasomes have been described, the best characterized being centered around Nlrp1, Nlrp3, ICE-protease activating factor (IPAF)/Nlrc4, and the HIN-200 members absent in melanoma 2 (AIM2) and IFN-γ inducible protein 16 (IFI16) [9]. In addition, the Nlrs Nlrp6 and Nlrp12 have also been suggested to assemble inflammasomes, although they also have been linked to regulation of MAP kinase and NF-κB signaling [12].

While they were originally described in immune cells, recent data show expression of inflammasome components in the CNS [13–16] and their activation by prototypical neurological insults such as amyloid-beta [13]. Limited data shows that inhibition of inflammasome components with antibodies against ASC or by using Nlrp3 knock-out mice protects against spinal cord injury [17, 18] and cuprizone-mediated demyelination [19]. In the peripheral nervous system (PNS), the role of IL-1 and especially the inflammasome is less well established. Therefore, we aimed to analyze expression of inflammasome components in the PNS and to evaluate their role in a model of acute peripheral nerve injury.

## Material and methods

### Mice work

All animal experiments were approved by the local ethics committee (University of Antwerp and University of Ghent) and conducted according to the guidelines of the Federation of European Laboratory Animal Science Associations (FELASA). In this study, wild type, caspase-1/-11 double knock-out, and ASC, Nlrp3, and Nlrp6 single knock-out mice were used [12, 20–22]. All mice had a C57BL/6 background, and either sex was used.

### Cell isolation

All primary cell isolations were conducted as described previously [23]. Briefly, primary Schwann cells were isolated from neonatal mouse sciatic nerve taken from P4–P6 pups from C57BL/6 mice [24, 25]. Neonatal animals were decapitated, and sciatic nerves were isolated. The peri- and epineurium of the sciatic nerves were removed, and the nerves were enzymatically and mechanically dissociated. The Schwann cells were plated on poly-l-lysin- and laminin-coated culture plates in DMEM/5 % horse serum supplemented with 10 μM cytosine b-D arabinofuranoside to remove mitotic cells. After 72 h, the medium was replaced with defined medium [26] and refreshed every 2 days. Motor neurons were isolated from the ventral part of spinal cord from E13 mouse embryos [27]. The ventral part of the spinal cord was dissociated both mechanically and enzymatically. Glial cells and motor neurons were separated by centrifugation in a 6.2 % OptiPrep gradient. Motor neurons were plated on poly-l-ornithine- and laminin-coated plates in NB medium (Invitrogen) supplemented with 2 % B27 supplement, 4 g/l glucose, 2 mM l-glutamine, and 50 ng/ml NGF. Sensory neurons were isolated from dorsal root ganglia (DRGs) from E13 mouse embryos [28]. The spinal cord was dissected, and DRGs were carefully removed. DRGs were enzymatically and mechanically dissociated, and cells were plated on poly-l-lysin- and laminin-coated dishes in NB medium supplemented with 2 % B27 supplement, 4 g/l glucose, 2 mM l-glutamine, and 50 ng/ml NGF. To obtain pure sensory neuron cultures, cycling every 2 days between the above-described medium and the same medium enriched with 1 % (*v*/*v*) FUDR (final 10 μM FdU and 10 μM uridine) was necessary. Peritoneal macrophages were isolated from adult mice that were injected with 3 % thioglycollate [29]. On day 4 after injection, mice were euthanized and the peritoneal cavity rinsed with ice-cold phosphate-buffered saline (PBS). Macrophages were collected and resuspended in RPMI with 1 % fetal calf serum (FCS). Erythrocytes, in suspension, were removed after 45 min. The remaining cells were kept in RPMI with 10 % FCS. The next day, dendritic cells in suspension were removed and macrophages were kept in culture.

### Induction of peripheral nerve injury

Axotomy experiments of the *Nervusischiadicus* (sciatic nerve) were conducted in 6- to 8-week-old C57BL/6 mice as previously described [23]. Briefly, mice were anesthetized with a single intraperitoneal injection of ketamine (Ketalar; Pfizer; 150 mg/kg) and xylazine (Rompun; Bayer; 10 mg/kg). An incision was made at the right thigh, and gluteal and hamstring muscles were carefully separated to expose the sciatic nerve. The sciatic nerve was transected or crushed, and the wound was closed by sutures. The contralateral (control) side was left untouched. For analgesia, bupronorphinum (Temgesic; Schering-Slough; 0.1 mg/kg) was injected after surgery. We first evaluated the effect of sham operation on the induction of several Nlrs, but did not find rigorous effects (data not shown).

### Intravenous injection of LPS

Lipopolysaccharide (LPS; TLR4-ligand) (Sigma; 10 mg/kg) was injected intravenously in 6- to 8-week-old C57BL/6 mice. PBS was injected in the control mice.

### RNA isolation and RT-qPCR

At 4, 24, 48, or 72 h after sciatic nerve transection, the mice were euthanized. The distal part of the transected *N. ischiadicus* and the contralateral control side were removed, snap frozen, and stored at −80 °C until use. The nerves were homogenized in TRIzol with a Potter-Elvehjem homogenizator, and small fragments were further homogenized by sonication. Total RNA was extracted using the RNeasy Lipid Tissue kit (Qiagen) according to the manufacturer’s protocol. DNase treatment was performed with TURBO DNase (Ambion), and cDNA synthesis was done with the Superscript III first strand synthesis system for RT-PCR (Invitrogen). RNA from cell populations that were isolated from the sciatic nerve was obtained using the RNEasy Plus Micro Kit (Qiagen) following the manufacturer’s instructions, and RNA integrity was assessed using a Bioanalyzer 2100 (Agilent). Using the Ambion WT Expression Kit, per sample, an amount of 50 ng of total RNA spiked with bacterial poly-A RNA positive controls (Affymetrix) was converted to double-stranded cDNA in a reverse transcription reaction. Real-time quantitative polymerase chain reactions (RT-qPCRs) were performed with 10 ng cDNA in SYBR Green I mix and run on a ViiA 7 Real-Time PCR System (Applied Biosystems). All PCR reactions were done in triplicate. Primers were designed making use of PrimerBank. Primer sequences are listed in Table 1. The RT-qPCR data were normalized according to the method described by Vandesompele et al. [30], by geometric averaging of multiple internal control genes. Processing of raw data and calculation of normalized relative quantities were done by using an improved version of the ΔΔCt method [31]. The mRNA expression levels are expressed relative to the basal condition (not-operated mice or not-injected mice). The two most stable housekeeping genes out of five for the different experiments were hydroxymethylbilane synthase (HMBS) and 60S ribosomal protein L13a (RPL13a).

**Table 1:** Primer sequences for RT-qPCR

Nlr- and inflammasome-related genes		
ASC	CAGCACAGGCAAGCACTCA	GGTGGTCTCTGCACGAACT
IPAF	AGGAATTCCAAGCTCACACC	ATCACCTGAAGCTCCACCTC
Casp1	GGGACCCTCAAGTTTTGCC	GACGTGTACGAGTGGTTGTATT
Casp11	AGGCTTTGCAGAGAAAAGACAC	CCCATACCTCAGTGAGAGATGT
Nlrp1a	TTAGATGAGCATGCCATTGC	ACTCCTGAAGACACAAGTGG
Nlrp2	AAGGAGCTAAAAGGCCAGAGG	TCTTTGGGTTACACAATGCCAG
Nlrp3	TGTGAGAAGCAGGTTCTACTCT	GGATGCTCCTTGACCAGTTGG
Nlrp4e	ATATCCAAGTAAGAAAAGCC	GAGAGCCTCCTCAGCAAACAC
Nlrp5	GAAAGCACAATGGGTCCTCCA	CTGACGCCTGTTCCACTTCT
Nlrp6	AGCTGAGAACGCTGTGTCG	AACTTGGGAACCCCGAAGC
Nlrp9b	CGAAAATCGAGAATTCTTCC	ACCTGTAGAAACAGGCTTAAC
Nlrp10	TCAAGACGCTGAAGTTCCACT	TGCTCCGTACATTGAAATCAGTT
NAIP1	TGCCCAGTATATCCAAGGCTAT	AGACGCTGTCGTTGCAGTAAG
NAIP2	GCTGTGGATTGAGTGTCTTAGAG	GTTCTCCCTCGAAGGAACTGC
NAIP5	TGCCAAACCTACAAGAGCTGA	CAAGCGTTTAGACTGGGGATG
NAIP6	AGCCACCAGCTATAAATGAGGA	CAGATTCCAGTACCCTTCACTGA
NOD1	GAAATTGGCTTCTCCCCTTC	ATAGGTCTCCTCCAGCAGCA
NOD2	CTCCACTGCCTCTGCCTTAC	GCAGCTCCAAGATGTTCTCC
Interleukin-1-family-related genes		
IL-1β	GCAACTGTTCCTGAACTCAACT	ATCTTTTGGGGTCCGTCAACT
IL-1α	CTGATGAAGCTCGTCAGGCAG	TGGTGCTGAGATAGTGTTTGTC
IL-18	GACTCTTGCGTCAACTTCAAGG	CAGGCTGTCTTTTGTCAACGA
IL-1ra	GCTCATTGCTGGGTACTTACAA	CCAGACTTGGCACAAGACAGG
Immune mediators		
MIP-1α	TTCTCTGTACCATGACACTCTGC	CGTGGAATCTTCCGGCTGTAG
TNF	CCCTCACACTCAGATCATCTTCT	GCTACGACGTGGGCTACAG
IL-6	TAGTCCTTCCTACCCCAATTTCC	TTGGTCCTTAGCCACTCCTTC
MCP-1	TTAAAAACCTGGATCGGAACCAA	GCATTAGCTTCAGATTTACGGGT
Housekeeping genes		
ACTB	GCTTCTAGGCGGACTGTTACTGA	GCCATGCCAATGTTGTCTCTTAT
B2M	ATGCACGCAGAAAGAAATAGCAA	AGCTATCTAGGATATTTCCAATTTTTGAA
HMBS	GAAACTCTGCTTCGCTGCATT	TGCCCATCTTTCATCACTGTATG
RPL13a	CCTGCTGCTCTCAAGGTTGTT	TGGTTGTCACTGCCTGGTACTT
TBP	TCTACCGTGAATCTTGGCTGTAAA	TTCTCATGATGACTGCAGCAAA

### Sorting of different cell populations from the peripheral nerve

Sciatic nerves were cut and digested with Liberase TM (0.02 mg/ml; Roche) and Dnase I recombinant (0.01 U/μl; Roche) for 30 min at 37 °C in RPMI. The cell suspension was filtered over a 100-μm nylon mesh. The cells were first stained with P75NTR-biotin and subsequently with Ly6C-FITC, SiglecF-Pe, CD11c-PeCy7, CD64-APC, MHCII-APCCy7, Ly6G-Alexa Fluor 700, CD11b-Pacific Blue, CD3-PeCy5, CD19-PeCy5, streptavidin-Pe Texas Red, and a live-dead marker conjugated to AmCyan. Schwann cells were isolated by cell sorting gating on FSC/SSC/singlets/P75NTR^+^/alive. Resident macrophages were isolated by cell sorting gating on FSC/SSC/singlets/CD11b^+^/SiglecF^−^/Ly6G^−^/CD3^−^/CD19^−^/Ly6C^−^/CD64^+^/MHCII^+^/alive whereas monocytes were isolated by gating on FSC/SSC/singlets/CD11b^+^/SiglecF^−^/Ly6G^−^/CD3^−^/CD19^−^/MHCII^−^/CD64^lo^/Ly6C^+^/alive. Cell sorting was performed on a FACSAria (BD Biosciences).

### Western blot analysis

For Western blot analysis, 5-mm segments distal to the transected *N. ischiadicus* and the contralateral control side were carefully removed, snap frozen, and stored at −80 °C until use. Protein lysates were prepared in E1A lysis buffer (1 % NP-40, 20 mM HEPES (pH 7.9), 250 mM NaCl, 20 mM β-glycerophosphate, 10 mM NaF, 1 mM sodium orthovanadate, 2 mM dithiothreitol, 1 mM EDTA, and a protease inhibitor cocktail) by homogenization in a Potter-Elvehjem homogenizator. Total protein concentration was determined by Bradford. Equal amounts of protein lysates (30 to 40 μg) were separated on NuPAGE gels, transferred to nitrocellulose membranes, and analyzed by immunoblotting. Briefly, membranes were blocked using blocking buffer (5 % milk in PBS containing 0.1 % Tween-20 or 5 % BSA in TBS containing 0.1 % Tween-20) and incubated overnight at 4 °C with a primary antibody. Secondary HRP-conjugated antibodies were used to visualize antibody signals on films using the ECL system (Thermo Scientific). As a positive control for caspase-1 antibody, the intestine of WT and caspase-1/-11-deficient mice that were given dextran sodium sulfate (DSS; 2 %; orally) was used. For the detection of IL-1β in Schwann cell cultures, Schwann cells were stimulated with LPS (2 μg/ml) for 4 h and ATP (5 mM) for the last 30 min. The proteins in the supernatant were precipitated using methanol and chloroform. Total protein samples were loaded on NuPAGE gels and processed as described above. Antibodies used were anti-Nlrp3 (Adipogen; Cryo-1), anti-IL-1β (R&D Systems; AF-401), anti-caspase-1 (Santa Cruz; sc-514), and anti-beta-actin (Abcam; A5441).

### ELISA

To determine cytokine levels in C57Bl/6 mice, segments 5 mm long (*n* = 4) distal to the injury were dissected and protein lysates were obtained in cell lysis buffer (E1A). IL-1β protein levels were determined in the protein lysates using enzyme-linked immunosorbent assays (ELISA) according to the manufacturer’s protocol (BD Biosciences) and with an anti-IL-1β antibody from eBiosciences (cat. no. 88-7013). The absorbance was recorded at 450 nm with a plate reader.

### Behavioral analysis

Recovery of the locomotor function after sciatic nerve injury was assessed by using the sciatic functional index (SFI). The SFI was calculated by using the formula adapted for mice by Inserra et al. [32]. Footprints were analyzed pre-operatively and every week until 7 weeks post-surgery. The behavioral analyses were done blind with respect to the identity of the animals. The SFI values of the knock-out mice were compared to the SFI value of the control group (wild type C57Bl/6 mice). Data on SFI are presented as mean ± SEM.

### Statistical analysis

For the different groups of animals included in the behavioral analysis experiments, the SFI values, before and after nerve injury, were compared using an ANOVA. Post hoc comparisons were made using the Bonferroni test. Comparisons of SFI values between groups were made using two-way repeated measures ANOVA followed by the Bonferroni test. All statistical analyses were performed using SPSS software.

## Results

### Basal expression of inflammasome components in the peripheral nervous system

While recent data reported on the expression of Nlr family members in spinal cord and brain [13–16], as to our knowledge, no data have been published on Nlrs in the PNS. Still, it has been shown that IL-1 can be produced by Schwann cells and can be found expressed in the sciatic nerve in conditions of chronic nerve injury [33–38]. We therefore decided to check the expression profile of the Nlr family in the sciatic nerve and in the most common cell types of the PNS. Primary Schwann cell, motor neuron, and sensory neuron cultures were obtained as described previously [23] and in the “Material and methods” section. The expression profile was compared to that of primary isolated peritoneal macrophages, known to express a broad range of immune receptors. The Nlr family members can be subdivided in different subfamilies according to their domain structure. All Nlrs are characterized by a central nucleotide-binding and oligomerization (NACHT) domain, flanked by C-terminal LRRs and an N-terminal caspase-recruitment domain (CARD) in the Nlrc subfamily (including IPAF and NOD1/2), a pyrin domain (PYD) in the Nlrp subfamily, or baculovirus inhibitor repeat (BIR) domains in the Nlrb or NLR family, apoptosis inhibitory protein (NAIP) subfamily [10, 39]. We analyzed the expression of 15 members coming from the three different subfamilies (Nlrb, Nlrp, and Nlrc) as well as the adaptor protein ASC using RT-qPCR. For the individual cell types three independent experiments and for the sciatic nerve five independent experiments were pooled.

**Fig. 1:**
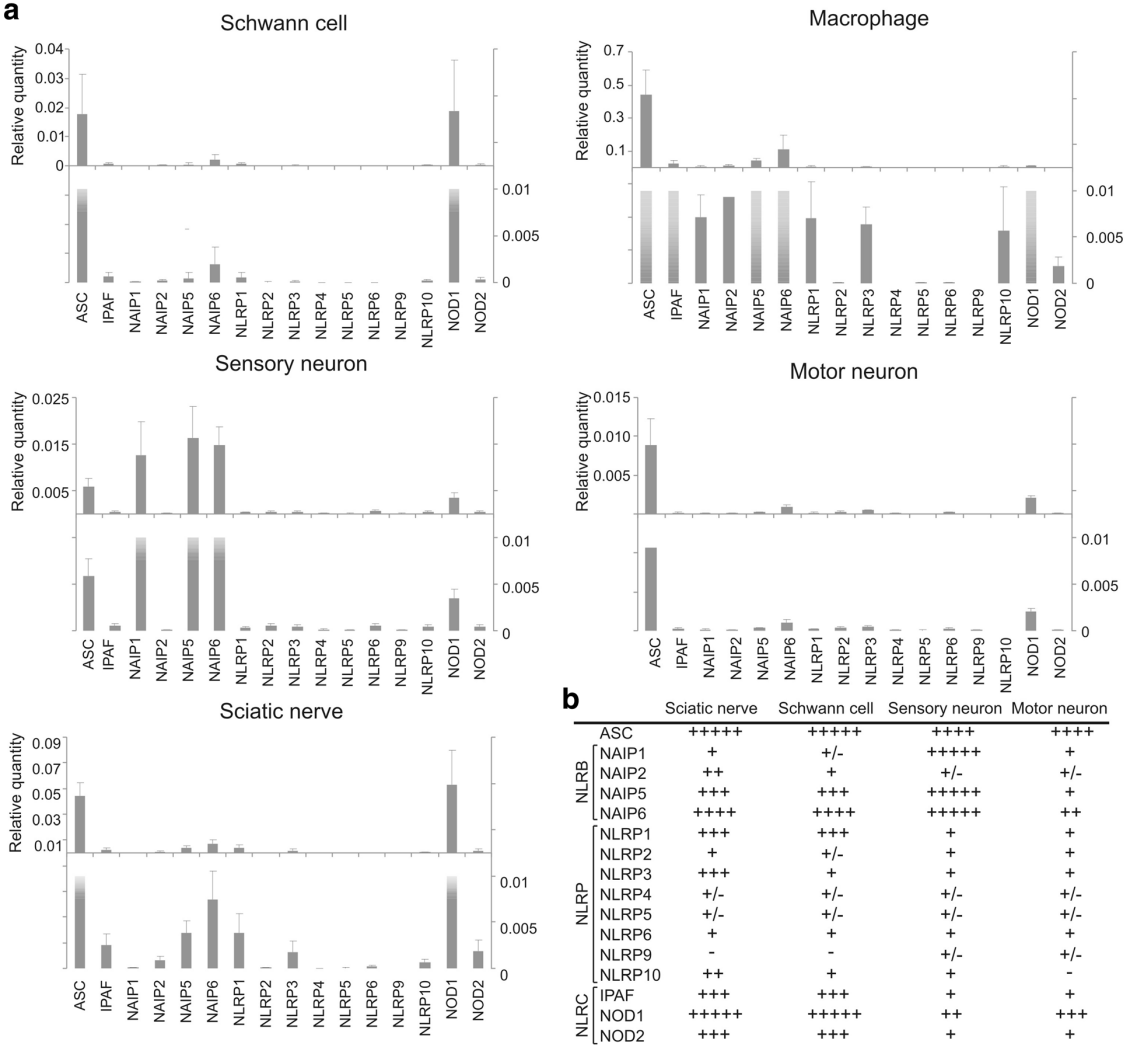
Nlr expression in primary Schwann cells, motor neurons, sensory neurons, peritoneal macrophages, and the sciatic nerve. **a** Nlr expression relative to the two most stable housekeeping genes out of five in the different primary cells, as determined by RT-qPCR. Note the different *Y*-axis scales used in the upper panel of each cell type. In the lower panel, the *Y*-axis is set to the same scale. In the graphs, the pooled data of three independent experiments is shown. **b** Ranking of Nlrs according to their relative expression levels in different PNS cell types as well as in the sciatic nerve, based on three to five independent experiments. *−* absent; *+/−* borderline detectable; *+*, *++*, *+++*, *++++*, *+++++* indicate low to very strong expression

As shown in Fig. 1a, b, several of the Nlrs tested in this study were expressed in the peripheral nerve and in the most common PNS cell types. The expression level was in general very low, as compared to macrophages (about 10–50×-fold less). Still, we could clearly detect cell-type-specific differences in the expression of Nlrs. Schwann cells expressed merely ASC, NAIP6, and NOD1. In sensory neurons, the Nlrb subfamily was overrepresented with major expression of NAIP1, NAIP5, and NAIP6 and lower expression of ASC and NOD1 (Fig. 1a, b). Motor neurons expressed very low levels of most Nlrs, and only ASC was clearly above detection limit. In the sciatic nerve, a compilation of those data could be seen and especially the Nlrb (NAIP) and Nlrc (IPAF/NOD) subfamilies and the adaptor protein ASC were highly expressed. In basal conditions, the Nlrp subfamily was less represented, although Nlrp1 and Nlrp3 were clearly detectable. Ranking of the different Nlrs according to their expression in the cell types or the nerve showed an almost identical expression pattern in Schwann cells and the peripheral nerve (Fig. 1b).

### Nlrs are differentially regulated in the nerve upon injury and LPS injection

**Fig. 2:**
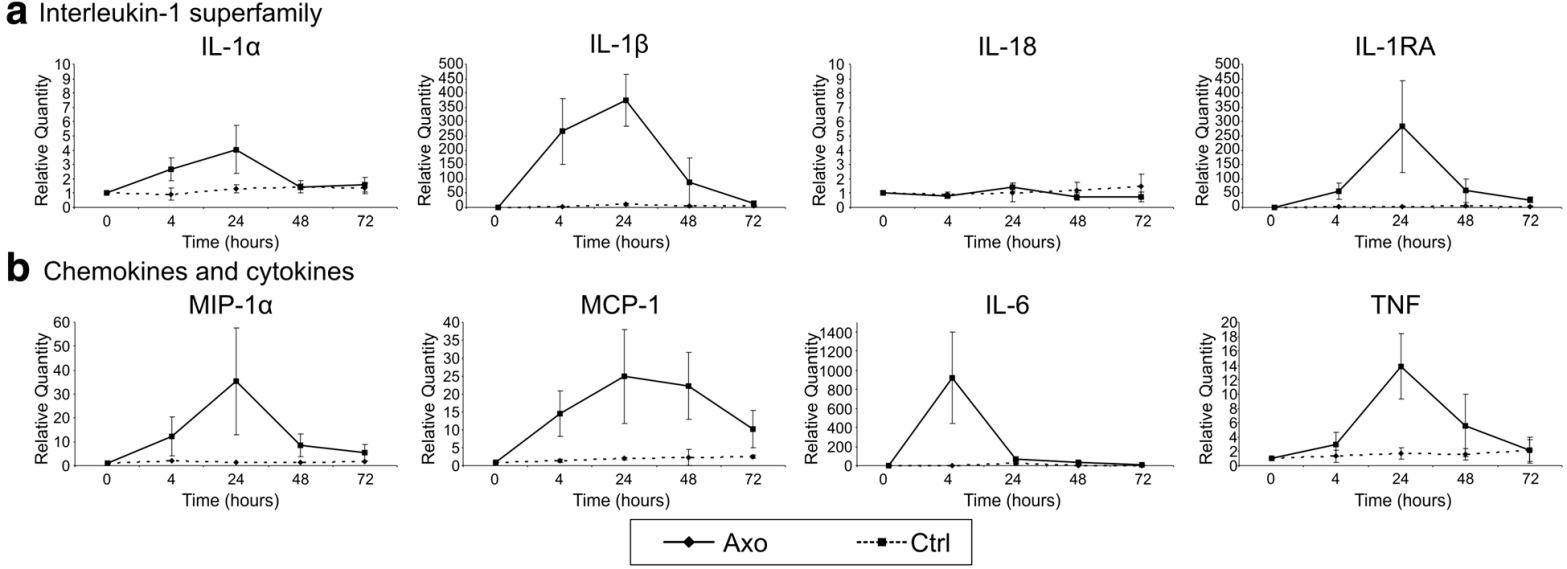
Induction profile of immune mediators upon acute injury of the sciatic nerve. **a** Induction of IL-1 family members upon injury of the sciatic nerve, as shown by RT-qPCR analysis. **b** Induction of chemokines (MIP-1α and MCP-1) and cytokines (IL-6 and TNF) at different time points upon injury of the sciatic nerve. Data is analyzed using the ΔΔCt method, and mRNA expression levels are expressed relative to the basal condition (0-h time point). The graphs show the pooled data of five independent experiments (mean ± SD)

Next, we addressed whether Nlrs are regulated in the peripheral nerve upon inflammation. Sterile injury was induced by axotomy of the sciatic nerve. At different time points after the injury, the distal segment of the sciatic nerve was isolated; total RNA was isolated, converted to cDNA, and analyzed by RT-qPCR. The contralateral side was used as a control. We first analyzed the transcriptional regulation of the most established members of the IL-1 superfamily: IL-1α, IL-1β, IL-18, and IL-1 receptor antagonist (IL-1ra). Both IL-1β and the antagonist were highly induced already 4 h after injury (Fig. 2a). IL-1α was only slightly induced and IL-18 was not induced at the transcriptional level (Fig. 2a). Several chemokines (macrophage inflammatory protein-1 alpha (MIP-1α) and monocyte chemoattractant protein-1 (MCP-1)) and cytokines (IL-6 and TNF) were taken along as a positive control for the inflammatory response, and all transcripts were markedly increased (Fig. 2b).

**Fig. 3:**
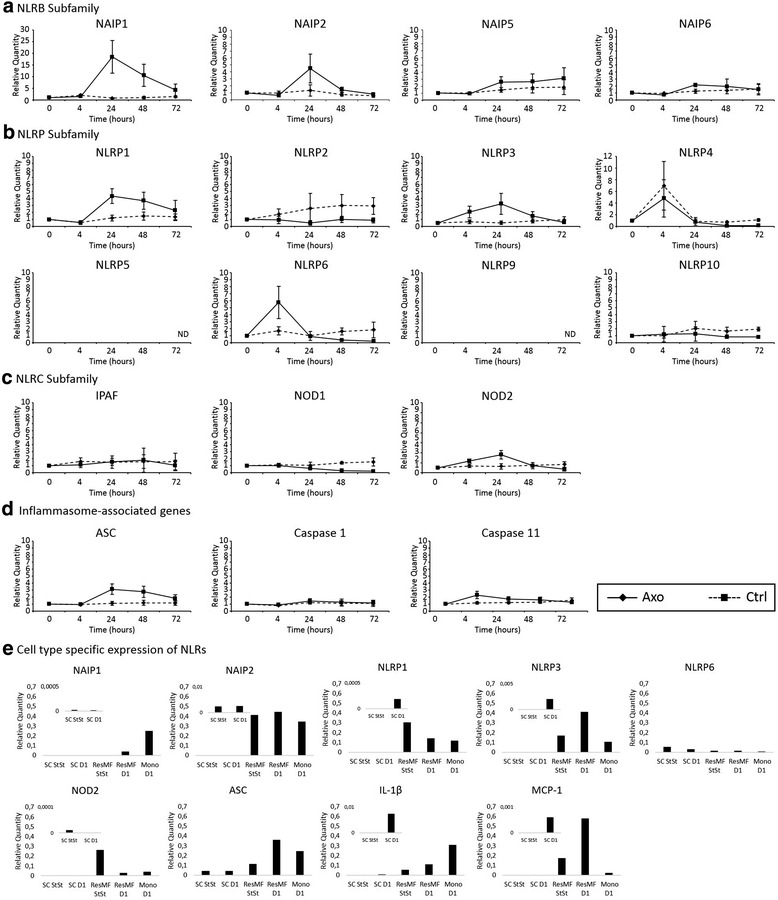
Induction profile of Nlrs upon acute injury of the sciatic nerve. Expression profile of Nlrb (**a**), Nlrp (**b**), and Nlrc (**c**) subfamily members during acute nerve injury. **d** Expression profile of several inflammasome components during acute nerve injury. Data is analyzed using the ΔΔCt method, and mRNA expression levels are expressed relative to the basal condition (0-h time point). The graphs show the pooled data of five independent experiments (mean ± SD). **e** Cell-type-specific expression of Nlrs in steady state and at day 1 post-injury in the peripheral nerve. Schwann cells, resident macrophages, and monocytes were sorted from the peripheral nerve, and expression of several inflammatory markers was determined by RT-qPCR analysis. *Mono D1* monocytes at day 1 post-injury, *ResMF D1* resident macrophages at day 1 post-injury, *ResMF StSt* resident macrophages at steady state, *SC D1* Schwann cells at day 1 post-injury, *SC StSt* Schwann cells at steady state, *ND* not detectable

Subsequently, we analyzed the expression profile of the Nlrs in conditions of peripheral nerve injury. Upon acute neurodegeneration, the Nlrb members NAIP1 and NAIP2 were strongly induced 24 h after surgical intervention, while NAIP5 and NAIP6 expression levels remained almost constant (Fig. 3a). Several members of the Nlrp subfamily—Nlrp1, Nlrp3, and Nlrp6—showed increased expression, with Nlrp3 and 6 already showing increased expression from 4 h post-surgery on. Nlrp3 levels remained high until 24 h after injury while Nlrp6 showed a sharper expression peak (Fig. 3b). Nlrp5 and Nlrp9 were borderline detectable over the time course of the experiments. The other Nlrp members did not differ from the contralateral control side (Fig. 3b). NOD2 became slightly induced 24 h upon injury, while NOD1 and IPAF did not increase in expression level (Fig. 3c). In addition, the transcripts of inflammasome-associated genes ASC, caspase-1, and caspase-11 were only moderately induced (ASC) or remained unaffected (caspase-1 and caspase-11) (Fig. 3d). There were no sex differences (data not shown). To find out which cell types contributed to the expression of these different Nlrs in the nerve, we sorted Schwann cells, resident macrophages, and infiltrating monocytes in steady state and at day 1 post-injury from the nerve. The purified cell populations were used for RT-qPCR analysis, and the results are shown in Fig. 3e. NLR molecules are mainly expressed in monocytes and resident macrophages and to a far lesser extent in Schwann cells. It seems that especially monocytes contribute to the observed induction of NAIP1, NAIP2, and Nlrp1 at day 1 after peripheral nerve injury, while resident macrophages induce the expression of NAIP1, Nlrp3, ASC, and MCP-1 1 day after injury. Schwann cells clearly show induction of IL-1β, MCP-1, and Nlrp1 and Nlrp3 upon peripheral nerve injury, but in general, their expression is lower compared to resident macrophages and infiltrating monocytes (Fig. 3e, inserts).

**Fig. 4:**
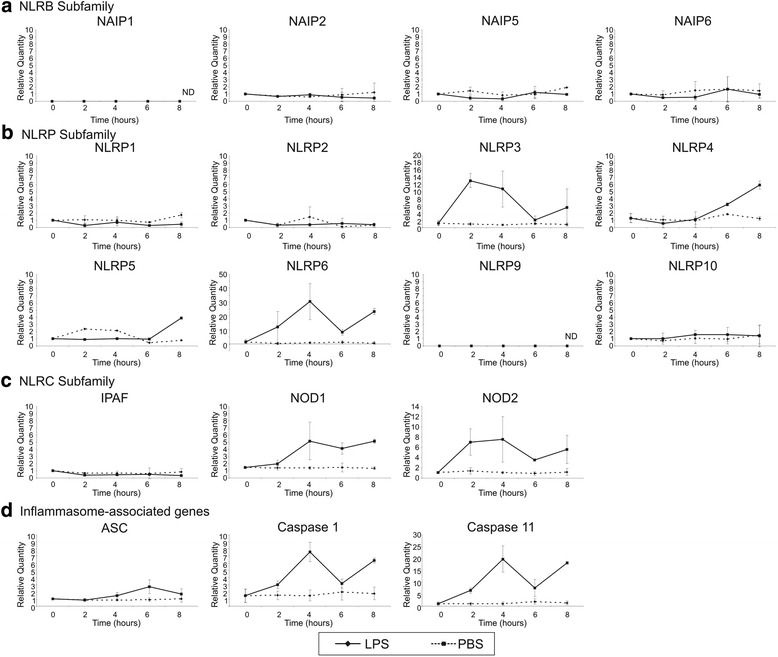
Regulation of Nlr expression in the peripheral nerve upon intravenous injection of LPS. Expression profile of Nlrb (**a**), Nlrp (**b**), and Nlrc (**c**) subfamily members upon injection of LPS. **d** Expression profile of several inflammasome components upon injection of LPS. Data is analyzed using the ΔΔCt method, and mRNA expression levels are expressed relative to the basal condition (0-h time point). The graphs show the pooled data of two to three independent experiments (mean ± SD). Note that scales of the graphs differ from the scales in Fig. 3. *ND* not detectable

To gain better insight into the role of the inflammasome and Nlrs in sterile inflammation of the peripheral nerve, the expression profile was compared to the profile induced upon a bacterial trigger. To this end, lipopolysaccharide (LPS) was injected intravenously and the expression profile of the same set of Nlrs was analyzed in the peripheral nerve. Intravenous injection of PBS was used as a control. Similar to injury-induced inflammation, LPS injection resulted in the high induction of Nlrp3, Nlrp6, and NOD2 in the sciatic nerve (Fig. 4b, c). The adaptor protein ASC was hardly affected (Fig. 4d). On the contrary, NOD1, Nlrp4, and the inflammatory caspase-1 and caspase-11 were clearly activated upon LPS injection. The NAIP genes and Nlrp1 remained very low (Fig. 4a, b). There were no sex differences observed (data not shown). This shows that a different set of Nlrs is induced in the nerve in the context of sterile versus bacterial inflammation, with a major role for the Nlrp and Nlrb family during sterile inflammation and for the Nlrp and Nlrc family during bacterial inflammation in the nerve.

### Nlrp6, but not Nlrp3, controls recovery after peripheral nerve injury

An important question remains whether the modulation of inflammasomes would control the recovery after peripheral nerve injury. As the inflammasome nucleated by Nlrp3 is the best characterized inflammasome in the context of sterile inflammation [40], and the expression of Nlrp3 was induced already 4 h after peripheral nerve injury, we assessed the functional recovery of knock-out mice for Nlrp3 using the sciatic function index (SFI). In addition, knock-out mice for Nlrp6 were also included in the experiment because this Nlr showed a marked transient increase immediately upon nerve injury and has been shown to play a prominent role in tissue repair and wound healing [41].

**Fig. 5:**
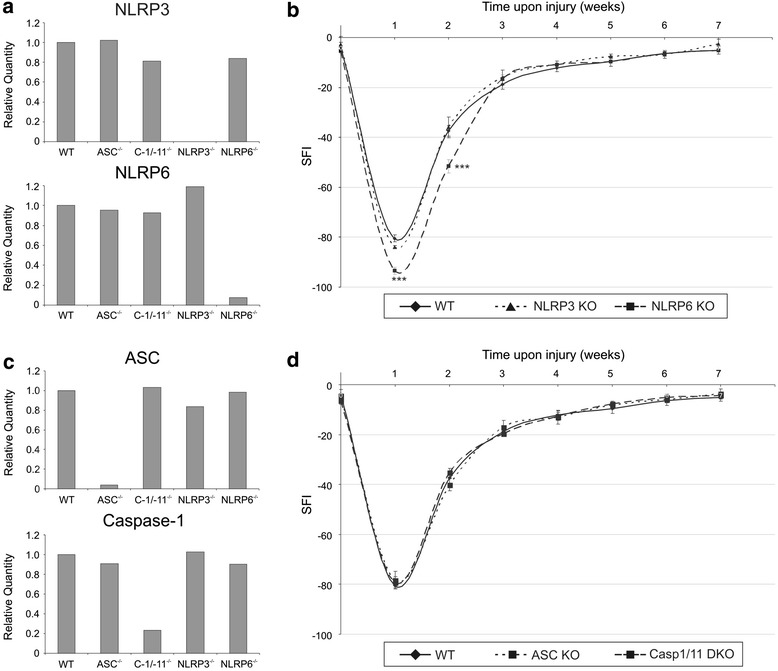
Functional recovery of inflammasome-deficient mice upon sciatic nerve injury. **a** Validation of the Nlrp3- and Nlrp6-deficient mice, using RT-qPCR. **b** Functional recovery of Nlrp3- and Nlrp6-deficient mice after peripheral nerve injury was determined by analyzing the SFI. Over a period of 7 weeks, Nlrp3 (*n* = 6) and Nlrp6 (*n* = 12) knock-out mice were compared to the control group (WT; *n* = 12). ***p ≤ 0.001 compared to the control group. **c** Validation of the ASC- and caspase-1/-11-deficient mice, using RT-qPCR. **d** Functional recovery of ASC- and caspase-1/-11-deficient mice after peripheral nerve injury was determined by analyzing the SFI. Over a period of 7 weeks, ASC- (*n* = 4) and caspase-1/11-deficient mice (*n* = 10) were compared to the control group (WT; *n* = 12)

Absence of Nlrp3 and Nlrp6 in the sciatic nerve was confirmed by RT-qPCR (Fig. 5a). Peripheral nerve injury was induced in wild type and knock-out mice by crushing the right sciatic nerve. Crush injury allows full recovery over time. The left sciatic nerve was left untouched and served as an internal control. Foot prints were measured prior to the surgery and every week thereafter for 7 weeks. There was no difference in the preoperative SFI values between the different groups included in this experiment. Compared to the wild type controls, Nlrp3 knock-out mice recovered at the same speed, indicating that the Nlrp3 inflammasome does not play a role in the functional recovery after peripheral nerve injury (Fig. 5b). In contrast, loss of Nlrp6 led to an exacerbated phenotype (Fig. 5b). The mice had a more dramatic drop in SFI immediately upon surgery (*p* < 0.001 compared to the control group at week 1 and week 2 after injury) and therefore needed more time to recover. At the end of the experiment, both WT and Nlrp6^−/−^ mice completely recovered though.

### Inflammasomes are dispensable for recovery after peripheral nerve injury

To characterize whether Nlrp6 regulated recovery after peripheral nerve injury through inflammasome activation, we analyzed mice upon crush injury lacking the central inflammasome adaptor protein ASC and double knock-out mice for caspase-1 and caspase-11. Absence of ASC, caspase-1, and caspase-11 in the sciatic nerve were confirmed by RT-qPCR (Fig. 5c). Peripheral nerve injury was induced in wild type and knock-out mice by crushing the right sciatic nerve. Foot prints were measured prior to surgery and every week thereafter for 7 weeks. Again, there was no difference in the preoperative SFI values between the different groups included in this experiment. In addition, it was clear that neither caspase-1/11 double knock-out mice nor ASC-deficient mice differed from control mice, indicating that IL-1β maturation does not seem to play a role in the functional recovery after peripheral nerve injury (Fig. 5d).

### Nlrp6 regulates ERK activation, but not IL-1β maturation, after peripheral nerve injury

**Fig. 6:**
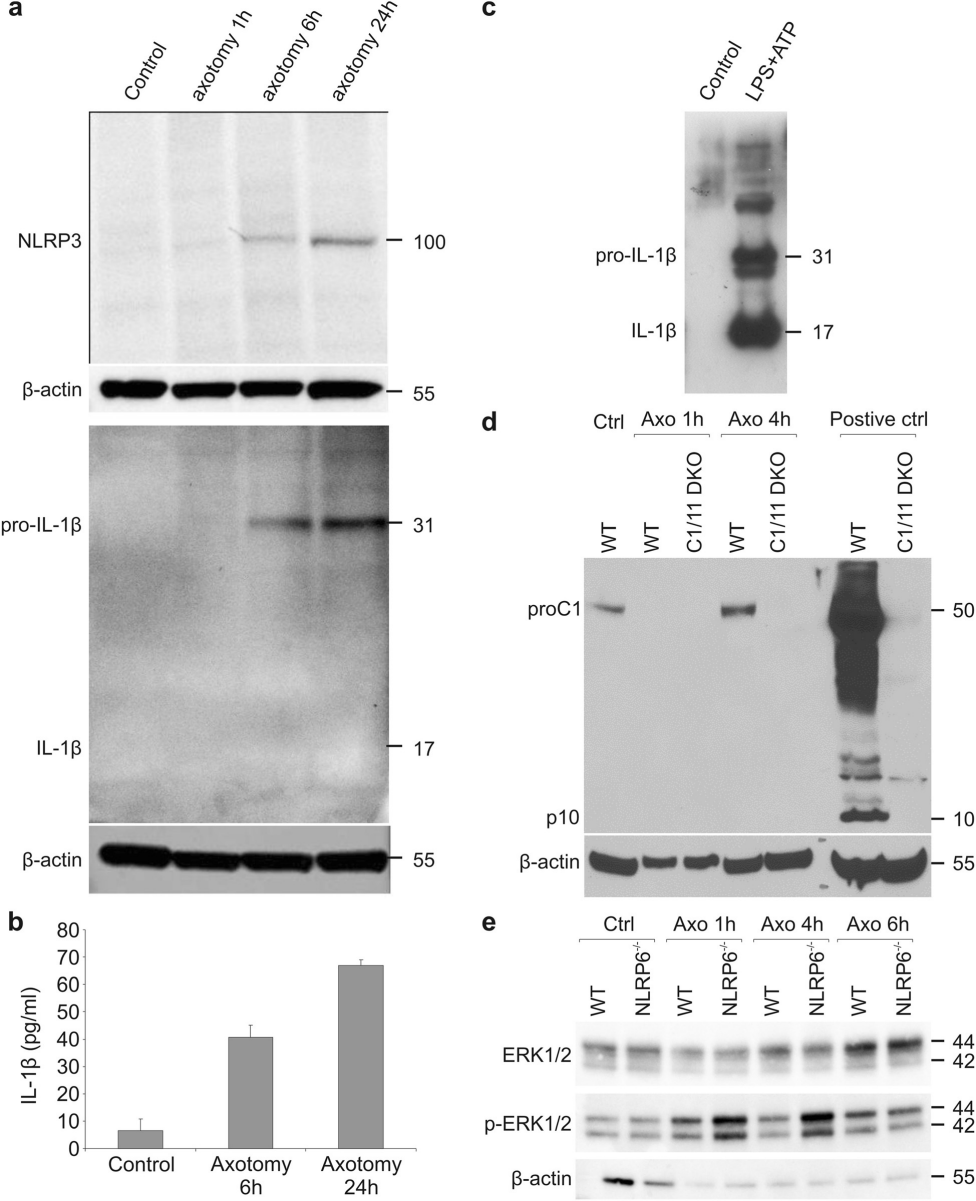
Activation of the inflammasome upon peripheral nerve injury. **a** Western blot analysis shows the induction of Nlrp3 and pro-IL-1β protein in axotomized sciatic nerves when compared to non-operated control nerves. **b** IL-1β protein expression in the injured sciatic nerve as determined by ELISA. **c** Western blot analysis of IL-1β secreted by Schwann cells stimulated with LPS and ATP. **d** Western blot analysis for caspase-1 protein in wild type and caspase-1/-11 knock-out mice. Intestine lysates of WT and caspase-1/-11-deficient mice that were treated with dextran sodium sulfate (DSS) (a known trigger for caspase-1 cleavage) were used as a positive control for the Western blot. **e** Western blot analysis shows increased activation of ERK1/2 upon peripheral nerve injury in Nlrp6^−/−^ mice compared to WT controls. For Western blot analysis, MW values expressed in kDa are shown at the right side of the blots. β-actin was used as a loading control

To check whether peripheral nerve injury is associated with IL-1β production at all, we analyzed expression of the whole signaling cascade at the protein level. Nlrp3 protein was not detectable in the sciatic nerve in steady state, but it was gradually induced over time upon peripheral nerve injury (Fig. 6a). The protein was slightly detectable at 6 h post-injury and was clearly induced 24 h post-injury, which confirmed the RT-qPCR data. A similar trend was observed in the production of pro-IL-1β, with a slight induction at 6 h and a clear induction at 24 h post-injury as shown by Western blot and ELISA (Fig. 6a, b). From Western blot analysis, it was clear that this represented mainly the pro-form of IL-1β, running at 31 kDa, while the mature form of IL-1β (running at 17 kDa) remained undetectable (Fig. 6a). As a positive control for Western blot analysis, Schwann cells were stimulated with LPS + ATP, which resulted in clear induction of mature IL-1β (Fig. 6c). We next addressed whether the inflammasome was activated upon peripheral nerve injury. Maturation of caspase-1, the hallmark of inflammasome activity, was not observed. As seen in Fig. 6d, the cleavage of pro-caspase-1 (45 kDa) to its mature form (10 kDa) could not be detected. In contrast, a control sample did show nice cleavage of caspase-1 (Fig. 6d). These results indicate that the inflammasome is not active upon peripheral nerve injury and may explain why mice lacking Nlrp3 and inflammasome components had a normal phenotype.

In a recent paper, Nlrp6 has been defined as a negative feedback molecule limiting excessive inflammation and ERK activation [12]. To confirm these data, we looked at ERK activation in the peripheral nerve upon axotomy in WT and Nlrp6^−/−^ mice (Fig. 6e). We could indeed observe more enhanced ERK phosphorylation in the Nlrp6^−/−^ mice, which was especially prominent at early time points. Therefore, while we could not detect a role for IL-1β for functional recovery of the nerve, we could see that overactivation of the inflammatory response aggravates peripheral nerve injury.

## Discussion

The role of IL-1β in the peripheral nervous system is still debated. Several studies claim increased expression of IL-1β in the damaged nerve upon sciatic nerve lesion or in models of chronic constriction of the sciatic nerve [33, 35, 37, 42, 43]. Recent data from the CNS showed the role of the P2X7 and P2X4 receptors in purinergic regulation of the inflammasome [18, 44]. In the PNS, hardly anything is known on how IL-1β is produced. From a functional perspective, many studies clearly link expression of IL-1β in the PNS with the development of neuropathic pain and spontaneous ectopic neuronal activity [33, 35, 45, 46]. Genetic studies demonstrated reduced mechanical allodynia in mice lacking the IL-1β receptor as well as in mice lacking both IL-1β and TNF [35, 43]. Despite this, loss of IL-1β did not lead to improved functional recovery in models of sciatic nerve injury but rather aggravated the phenotype [35]. This was confirmed by several other studies claiming a neuroprotective role for IL-1β in sciatic nerve regeneration [36, 38]. IL-1β would be required for promoting Schwann cell migration, extracellular matrix remodeling, and neurite outgrowth [36, 38].

In the present study, we dissected the role of several key components of the inflammasome, the molecular platform needed for IL-1β processing, on functional recovery in a mouse model of sciatic nerve crush. Since no data were available yet on the expression profile of different inflammasome components in the PNS, we first set out on a detailed expression study of several Nlrs. In basal conditions, predominantly members of the Nlrb and Nlrc family are represented, while hardly any Nlrp family members could be detected. Peripheral nerve injury led to a strong increase of several Nlrp (NALP) and Nlrb (NAIP) family members, while the Nlrc (NOD and IPAF) subfamily was not affected. In the context of bacterial infection, especially the Nlrc and Nlrp family were induced, while this time the Nlrb family remained unaffected, showing distinct expression profiles of Nlr family members in the context of sterile versus bacterial inflammation. Despite the elevated expression of all essential components of the Nlrp1 and/or Nlrp3 inflammasome, we could not demonstrate release of mature IL-1β upon peripheral nerve injury, in contrast to earlier studies [33, 35, 37, 42, 43]. However, a closer look at the techniques used in these studies (RT-qPCR, ELISA, or immunohistochemistry) learned that most of them rely on techniques that do not allow making a distinction between inactive pro-IL-1β and mature active IL-1β. Antibodies available for ELISA and immunohistochemistry detect both the pro-form and mature IL-1β (see Fig. 6a, b and [47]), and the only validated technique to date to unambiguously demonstrate the presence of active IL-1β is to show Western blot data of the mature form running at 17 kDa. As to our knowledge, this was only convincingly included in the study by [35] which did observe expression of mature IL-1β in the sciatic nerve upon ligation. While we could detect mature IL-1β release from cultured Schwann cells upon LPS stimulation (Fig. 6c), we were never able to detect any major release of mature IL-1β upon sciatic nerve injury.

In line with these findings, we also did not observe any improvement or worsening of sciatic nerve functioning upon deletion of *ASC*, *Nlrp3*, or *caspase-1/-11*. As such, we could not conclude upon any essential role for the inflammasome during acute nerve injury in the PNS. An earlier study making use of IL-1β/TNF single and double knock-outs did show reduced mechanical allodynia and impaired recovery in a similar model of partial nerve ligation [35]. At the moment, we cannot reconcile these findings with our data as one would suspect that the caspase-1/-11-deficient mice should have a similar defect as the IL-1β-deficient mice (although caspase-1-independent cleavage mechanisms for IL-1β have been suggested as well [8]). Still, it should be noted that in the Nadeau paper the defect was especially pronounced in the double IL-1β/TNF knock-out, showing that both cytokines could compensate for each other.

In the CNS, the devastating role of IL-1β in acute neurodegeneration was convincingly demonstrated [1, 5, 6]. A major difference between acute injury in the CNS and the PNS, however, is the induction of a predominantly M1-polarized versus a predominantly M2-polarized response, respectively [48]. Whereas the pro-inflammatory M1 responses are typically associated with the induction of cytokines such as TNF and IL-1β, the inflammation-resolving M2 response is rather associated with the presence of anti-inflammatory cytokines [49]. It has been demonstrated that in macrophages polarization towards the M2 state does not affect their ability to induce pro-IL-1β in response to several triggers. However, ATP no longer leads to the induction of caspase-1 cleavage or mature IL-1β release in M2-polarized macrophages and this despite the presence of a fully intact Nlrp3 inflammasome [47]. This is very similar to what we observed during sciatic nerve injury and might explain why we do not see any effect of inflammasome loss on sciatic nerve recovery. The authors suggest that the uncoupling of ATP-mediated P2X7 stimulation to caspase-1 activation is part of a major switch to induce resolution of inflammation.

While in humans 22 Nlr family members have been described and in mice up to 34, only for a few of them their in vivo function has been unraveled [9]. In the peripheral nerve, a very transient expression of Nlrp6 could be noticed upon nerve injury. Nlrp6 has been shown to play critical roles in defense against infection and tumorigenesis and in maintaining intestinal homeostasis and a healthy, equilibrated intestinal microbial flora [12, 41, 50, 51]. The latter function has been suggested to be controlled by regulating IL-18 levels in an inflammasome-dependent manner [52]. Nlrp6 regulates the goblet mucus secretion [53], and self-renewal of the intestinal epithelium and its deficiency leads to aberrant wound healing, promoting colitis-associated colon carcinogenesis [41]. Recent studies showed that Nlrp6 is a negative regulator of the mitogen-activated kinase (MAPK) and canonical nuclear factor-kappa B (NF-κB) pathways [12]. Deficiency of Nlrp6 leads to resistance against infection with bacterial pathogens like *Listeria monocytogenes*, *Escherichia coli*, and *Salmonella typhimurium.* Upon infection, the mice show increased monocyte and neutrophil numbers and enhanced levels of cytokines and chemokines. This latter function is non-inflammasome dependent [12]. Based on its function in tissue repair and wound healing, we decided to look at the role of Nlrp6 in sciatic nerve recovery upon crush injury. In contrast to the *ASC*^−/−^, *Nlrp3*^−/−^, and *Casp-1/-11*^−/−^ mice, we did notice strongly decreased nerve function immediately upon nerve injury. The mice therefore recovered more slowly, although from 3 weeks post-surgery on they regained similar nerve function capacity. Confirming the data from [12], this was associated with enhanced levels of ERK phosphorylation and occurred independently from the inflammasome. Nerve injury is known to cause ERK activation [54, 55]. This is associated with increased pain sensation [56, 57] but at the same time also leads to Schwann cell dedifferentiation, which is needed for optimal repair [55, 58]. As such, the enhanced activation of ERK in the absence of *Nlrp6* could explain the decreased sciatic nerve function immediately upon the surgery, while later on it might actually help and even stimulate nerve recovery.

## Conclusion

In conclusion, our study shows a major role for Nlrp6 in regulating sciatic nerve recovery. We further show that this occurs independently of inflammasomes and of the production of mature IL-1β. This could well fit with the predominant M2 environment associated with sciatic nerve injury, which has been shown to impede caspase-1 activation and IL-1β release. On the contrary, loss of *Nlrp6* exacerbates loss of nerve function upon crush injury, which was associated with increased MAPK activation.
